# A Closer Look: Who Are Cancer Caregivers in 2025?

**DOI:** 10.1002/pon.70195

**Published:** 2025-06

**Authors:** Leah E. Walsh, Allison J. Applebaum

**Affiliations:** Brookdale Department of Geriatrics and Palliative Medicine, Icahn School of Medicine at Mount Sinai, New York, NY, USA

**Keywords:** cancer, caregiver burden, caregiving, psycho-oncology, supportive care

## 1 | Introduction

When a patient is diagnosed with cancer, it greatly affects not only their daily life, routines, goals, and hopes for the future, but those of the family and friends who support them during and after their cancer treatment. Caregivers are intimately intertwined with patients during all stages of the cancer journey: from understanding the implications of diagnosis and treatment options to managing the logistics of scans and care delivery, and the unpredictability of restaging, cancer progression, survivorship, and end-of-life care transitions. Caregivers provide immense support regarding gathering and understanding cancer-related information, assisting with medical or nursing tasks, and transportation, in addition to providing emotional support as patients cope with the threat to their life that cancer represents. They advocate for the patient with the healthcare team, navigate the uncertainty of cancer survivorship with the patient, and make plans for future care as patients near the end-of-life and caregivers face new realities in bereavement.

Understandably, caregiving is a significant stressor. Caregiving can be perceived as an intense struggle; an experience of meaning, purpose, and growth; and most often, some combination of both positive and negative emotions [1]. Caregivers experience burden, a broad term for the ways in which caregiving impacts the caregiver’s life—emotionally, physically, financially, spiritually, and in their daily activities. However, caregivers experience more than just an omnibus burden—their distress can lead to significant symptoms and psychopathology. Anxiety and depressive symptoms are common, with pooled prevalences across studies suggesting that around 46% and 42% experience significant anxiety and depressive symptoms, respectively [2, 3]. In addition to patients, caregivers can experience a fear of cancer recurrence that infiltrates their daily life with worries and “what-if’s,” an area that is gaining recognition in research. Caregivers can also experience symptoms of posttraumatic stress disorder after witnessing life-threatening events [4]. Moreover, the compounding suffering of caregiving may lead to a loss of meaning, with about 30% of caregivers reporting some level of existential distress [5]. The psychological and emotional challenges of caregiving are undoubtedly profound and call for more systematic screening and intervention.

Until recently, the science of cancer caregiving was in its infancy, with initial studies beginning in the early 2000s and more recent dedication to exploring the experience of providing care for someone with cancer [6]. Cancer caregiving researchers share the collective goal to design effective interventions and programs that are implemented in practice to alleviate suffering. In order to do so, however, we must first understand who cancer caregivers are.

Caregivers are often thought to be spouses who take on the caregiving role for their partner, or parents caring for a child with cancer. Over the past decade, however, it has become evident that caregiving relationships are much more diverse. Caregivers can be adults who may be caring for their own children and parents simultaneously, named the “sandwich” generation [7]. Caregivers can be partners early on in their romantic relationship. Caregivers can be siblings. Caregivers can be friends or neighbors. While there are benefits to exploring the unique experiences of particular caregiving relationships, when researchers limit inclusion for caregiving-related research to specific caregiving relationships (e.g., spousal or romantic partners), findings will undoubtedly limit our understanding of how other caregiving dynamics impact caregiver mental health challenges, financial toxicity, communication with the healthcare team, and advance care planning.

The caregiving literature overwhelmingly represents non-Hispanic white caregivers, with a firm call to focus on the needs of diverse caregivers and create culturally and linguistically responsive support [8]. Cultural and familial dynamics and expectations significantly impact who in a family is designated as the caregiver, as well as the caregivers’ perception of their role, communication during treatment, and their own personal meaning of caregiving [4, 9]. Moreover, people take on caregiving roles across the lifespan: older adults, adults, young adults, and those emerging into adulthood can all be caregivers grappling with their own responsibilities, goals, and wishes. However, there is limited representation of older and younger caregivers in research. The lack of representation of younger caregivers is a critical omission given that a large number (at least 24% of caregivers in the United States) are between 18 and 34 years old [10]. Some studies have even documented children and adolescents who often complete caregiving tasks within a family without the recognition of being a “caregiver,” [11] suggesting the presence of an understudied area of cancer caregiving.

As the immense burden of cancer caregiving has gained increased recognition and focus in clinical research, interventions have been adapted and tested to mitigate caregiver burden and distress. These interventions are frequently based in cognitive behavioral, acceptance and commitment, and meaning-centered therapies and have documented effects in reducing symptoms such as anxiety, depression, posttraumatic stress, and more general forms of burden [12, 13]. Disseminating clinical research to standard clinical practice is a challenge within and outside of psycho-oncology. However, without infrastructure on the ground dedicated to caregiver support, such interventions will never reach those caregivers who need them. As a result, there is new momentum to develop caregiver support programs within cancer centers, a movement that is supported by the recent Caregiver Clinical Services Toolkit published by the American Cancer Society [14]. This toolkit provides an in-depth overview of steps to take to create and maintain clinical services for cancer caregivers, with resources to support timely and robust mental health services for caregivers experiencing distress.

While infrastructure for cancer caregivers is crucial for future research and clinical programs, identifying and understanding the needs of the cancer caregiver begins on a much more personal level (Table 1). Healthcare professionals who work in oncology and psycho-oncology meet and speak to caregivers every day, meaning that there are daily opportunities to intervene on caregiving distress. Asking patients about their support networks, and asking their caregivers about how they support the patient, is a great first step to understanding those caregivers who are a vital extension of the healthcare team. A simple question of “How are you doing?” and “What is one thing that I can help you with?” may be the difference between a caregiver feeling validated - a caregiver being connected to some sort of support service - or not. Caregivers may present in medical appointments as distressed, or they may mask their distress behind organization and determination. Regardless of how they present, a simple question to caregivers can have immense downstream effects in terms of their own, as well as the patient’s, health and wellbeing. As clinicians seek to deliver the highest quality cancer care across settings, engaging and building relationships with patients’ caregivers provides us the opportunity to know more, do better, and ensure we are responding to the needs of all involved.

## Figures and Tables

**TABLE 1 |:** Closing out: Key takeaways about cancer caregivers.

1.	Many people in a patient’s life can be their caregiver. It is not always the spouse or parent who may assume that role, and multiple people may contribute to caregiving together. Thorough assessment of the patient’s support network and caregiving team can bolster care.
2.	Cancer caregiving and witnessing the patient’s medical and emotional suffering can lead to anxiety, depression, posttraumatic stress, and existential suffering. Simply asking caregivers how they are doing can be a powerful, yet brief, intervention and can help you connect them to appropriate resources. Measuring these constructs in clinical research is imperative to understanding their distress and designing interventions and programs that mitigate psychopathology and support caregivers in their responsibilities.
3.	Caregivers who are significantly distressed and exhibiting symptoms that impact their ability to provide care to the patient, or to complete their many other responsibilities, including those to themselves, may benefit from targeted psychotherapeutic support.
4.	Research that focuses on healthcare communication, prognostic understanding, treatment decision-making and utilization needs to include a caregiver perspective as they are a vital member of the healthcare team.

## Data Availability

Data sharing not applicable to this article as no datasets were generated or analyzed during the current study.
